# A Computational Predictor of Human Episodic Memory Based on a Theta Phase Precession Network

**DOI:** 10.1371/journal.pone.0007536

**Published:** 2009-10-23

**Authors:** Naoyuki Sato, Yoko Yamaguchi

**Affiliations:** 1 Department of Complex Systems, School of Systems Information Science, Future University–Hakodate, Hakodate, Hokkaido, Japan; 2 Laboratory for Dynamics of Emergent Intelligence, RIKEN Brain Science Institute, Wako-shi, Saitama, Japan; Newcastle University, United Kingdom

## Abstract

In the rodent hippocampus, a phase precession phenomena of place cell firing with the local field potential (LFP) theta is called “theta phase precession” and is considered to contribute to memory formation with spike time dependent plasticity (STDP). On the other hand, in the primate hippocampus, the existence of theta phase precession is unclear. Our computational studies have demonstrated that theta phase precession dynamics could contribute to primate–hippocampal dependent memory formation, such as object–place association memory. In this paper, we evaluate human theta phase precession by using a theory–experiment combined analysis. Human memory recall of object–place associations was analyzed by an individual hippocampal network simulated by theta phase precession dynamics of human eye movement and EEG data during memory encoding. It was found that the computational recall of the resultant network is significantly correlated with human memory recall performance, while other computational predictors without theta phase precession are not significantly correlated with subsequent memory recall. Moreover the correlation is larger than the correlation between human recall and traditional experimental predictors. These results indicate that theta phase precession dynamics are necessary for the better prediction of human recall performance with eye movement and EEG data. In this analysis, theta phase precession dynamics appear useful for the extraction of memory-dependent components from the spatio–temporal pattern of eye movement and EEG data as an associative network. Theta phase precession may be a common neural dynamic between rodents and humans for the formation of environmental memories.

## Introduction

Hippocampal place cells that are selectively activated by a specific portion of the environment are known to synchronously fire with local field potential (LFP) in the theta band (4–8 Hz) during locomotion. O’Keefe and Recce [1] reported an interesting relationship between place cell firing and LFP theta; phases of place cell firing to LFP theta gradually advance as the rat passes through the place field. Multi-unit recording findings further demonstrated that the individual place cells show different phase precession patterns [2]. Since the time scale of the phase difference of two place cell firings in a neighboring place field agrees with a time window of spike time dependent plasticity (STDP) [3], the phase precession pattern has been suggested to contribute to the synaptic plasticity in the hippocampus [2]. Computational studies have further demonstrated advantages of theta phase precession in the formation of sequence memory [4], [5], [6], [7], spatio-temporal patterns [8], cognitive maps [9] and goal-directed navigation in the environment [10]. Theta phase precession is considered a key mechanism of memory formation in the rodent hippocampus.

In the primate hippocampus, LFP theta appears intermittently [11] and place cells also have a firing rate in the theta range [12], [13], [14]. Although primate theta phase precession has not been evaluated, the same dynamics of theta phase precession are shown to have a computational advantage in the formation of object–place associative memory [15] that is a typical hippocampus-dependent memory in humans [16], [17], [18], [19]. In the computational model of object–place memory, input sequence is given by visual saccades in relation to a ’view cell’ property [20] where hippocampal units are selectively activated by eye fixation in the environment, and by both object and scene information in the central and peripheral visual field respectively in relation to the anatomical organization of the parahippocampal region [21]. The input sequence is translated to a phase precession pattern at the entorhinal cortex, is transmitted to the CA3 region, and is stored into unidirectional connections according to STDP. Surprisingly a hierarchical cognitive map representing object-scene associations by asymmetric connections is formed in a several second encoding period. Such memory structure is not a simple trace of input sequence but organizes individual object-scene associations into the whole object arrangement similar to a human cognitive map [22]. The model explains the neural mechanism of real-time environmental memory formation in humans.

According to the model of object–place memory with theta phase precession, recall performance is expected to be associated with electroencephalography (EEG) theta power and eye saccades during encoding, thus the prediction was evaluated in human experiments. First, the scalp EEG theta power during memory encoding significantly correlates with the subsequent recall performance [23]. The evidence also corroborates EEG results of word memory tasks [24], [25]. Second, the scalp EEG theta power is also coherent to saccade rate in relation to the subsequent recall performance [26]. The EEG theta and saccades cooperate during memory encoding, as predicted by the model. Third, a simultaneous EEG and functional MRI measurement further showed that the scalp EEG theta is correlated to the parahippocampal BOLD signal [27]. Although the scalp EEG might not directly detect the hippocampal LFP activities, the scalp EEG theta is expected to be a part of the global EEG theta network combining several brain regions during memory encoding.

In this paper, we evaluated the human theta phase precession by using a theory-experiment combined analysis. In the analysis, experimental data of human object–place memory recall were compared with the individual hippocampal network simulated by theta phase precession of human experimental data, eye movement and scalp EEG data during encoding. If similar dynamics to theta phase precession exist in the human brain, the individual hippocampal network should have the ability to predict human subsequent recall, otherwise the model of human phase precession is rejected.

## Results

### 2.1 Simulated memory with theta phase precession

**Figure 1:**
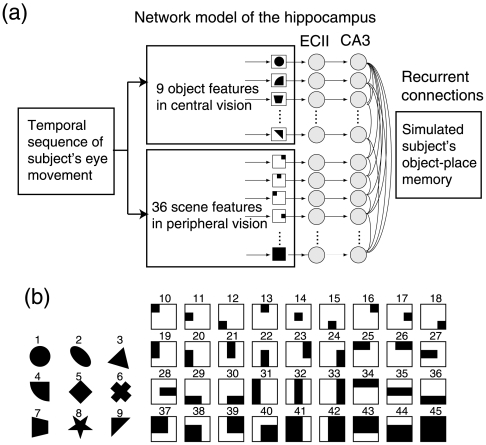
Basic structure of the model. (a) The model consists of an input layer, the ECII layer and the CA3 layer. Each layer includes 45 units. The eye movement sequences translated to the model input according to 9 object and 36 scene features. The input sequence is translated to theta phase precession pattern in the ECII layer, transmitted to the CA3 layer, and stored into connection weights according to a spike timing-dependent plasticity (STDP). The resultant associative network is expected to simulate subject’s memory. (b) Nine object and 36 scene features used in the model.

**Figure 2:**
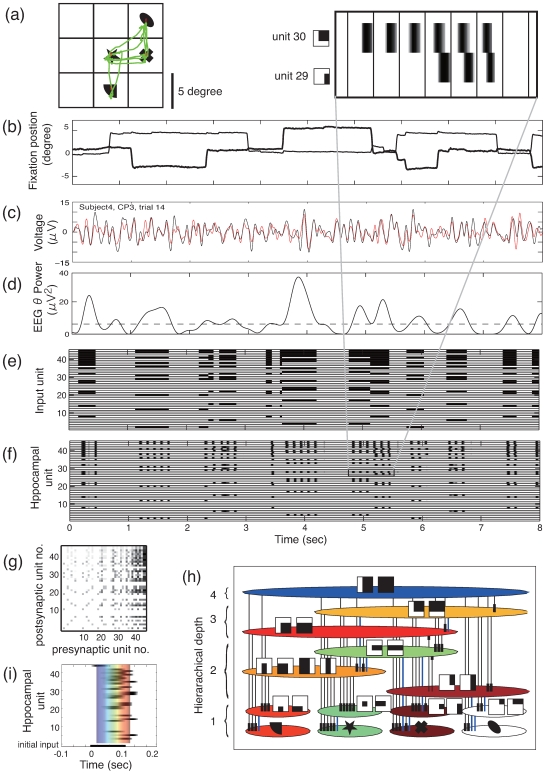
An example of the computational model–human experiment combined analysis. (a) Visual stimulus and eye movement during an 8 sec-encoding period (subject 4, trial 14). (b) Horizontal and vertical eye fixation on a location in the screen shown by thin and thick lines, respectively. Time is on the horizontal axis and the x and y positions on the screen are on the vertical axis. (c) Raw and ocular artifact corrected EEG data at CP3 electrode, shown by red and black lines, respectively. (d) EEG theta power (7 Hz) of the ocular artifact corrected EEG data. (e) Input sequence of the model. (f) ECII unit activation showing theta phase precession. (g) Resultant CA3 connection weights. (h) Connection weights represented by equivalent graphs. The nodes indicate subpopulations of units receiving the same visual input sequence. Lines show the connection weights between nodes, where the red line denotes the direction of the connections. Numbers at the side of the node indicate the number of object that activates the units in the node. (i) A recalled sequence initiated by a partial scene unit activation.

Fig. 1 shows the model of phase precession used in the analysis where subjects’ visual experience was stored into the connection weights in the CA3 network. Fig. 2 shows subjects’ eye movement and EEG data and temporal evolution of the unit activities in the model corresponding to these experimental data. Figure 2a and 2b show eye movement data during 8-sec encoding, where quick changes of eye positions correspond to eye saccades and stable eye positions correspond to eye fixation. Figures 2c and 2d indicate raw EEG data at the CP3 electrode and its wavelet power in the theta band (7 Hz), respectively. Figure 2e shows the input sequence of the model where the eye position at each time is encoded by given visual features (Fig. 1b) during a high EEG theta power period. The ECII layer receives the input sequence and translates it into the theta phase precession pattern (Fig. 2f). In theta phase precession, the “earlier-later” phase relationship of units represents the time difference of the onset time of these units’ input.

**Figure 3:**
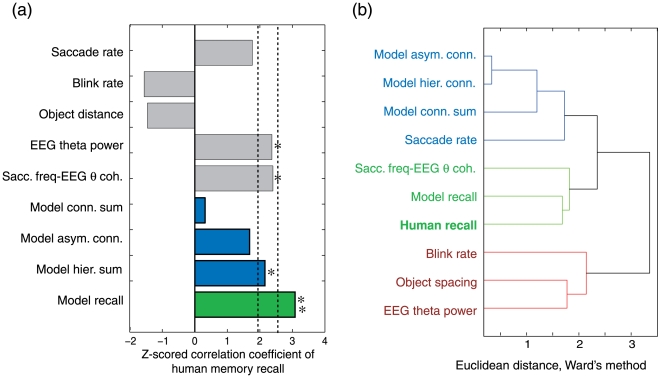
Results of statistical analysis. (a) Z-score of correlation coefficient between each predictor and subjects’subsequent recall. (b) Dendrogram of clustering using Ward’s method. Three clusters are found, where the computational and subject’s subsequent recalls appear in the same cluster.

The CA3 layer receives an input sequence encoded by phase precession, and stores it into connection weights according to the STDP. Figure 2g shows a resultant CA3 connection matrix after one trial 8-sec encoding, where the difference of connection weights along a diagonal line indicates asymmetric connections. In Figure 2h, the structure of the asymmetric connections is shown by an equivalent graph of the connection matrix, where each node represents a population of units with the same input sequence, each line with an arrowhead denotes directional connections, and numbers at the side of nodes represent overlap of the node units (i.e. a number of objects that could activate the node units). For example, a node marked by asterisk is only activated by ‘star’ and ‘cross’ objects and the overlap of the node is two. In the graph, directions of asymmetric connections are always found to appear from greatest to least overlap of the node units (from the top to the bottom in the graph). This structure is called a hierarchical cognitive map of object-place associations [15]. The number of nodes is restricted by the number of combinations of objects (i.e. maximum number of 15 in the case of four objects), where the usage of a spatially continuous receptive field (see, Fig. 1b) results in smaller number of nodes as shown in the graph. The structure of the network was evaluated by three predictors, the connection weights sum, 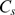, the asymmetric connection weight sum, 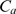, and the hierarchical connection weight sum, 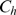. Spearman’s rank-sum correlation between the predictor and subjects’ subsequent recall performance was calculated. In the results shown in Figure 3a, the hierarchical connection weights sum, 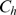, only showed a significant correlation with the subsequent recall performance (r = 0.1107, Z = 2.0662, p = 0.0388<0.05), while other predictors, 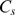 and 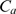, were not significant (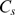: r = 0.0259, Z = 0.3718, p = 0.7100 (n.s.); 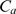: r = 0.0882, Z = 1.6825, p = 0.0925 (n.s.)). 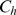 is considered to reflect a spatio-temporal pattern of eye movement orbit that is stored by theta phase precession. 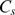 and 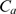 are considered to dominantly reflect fixation duration and unidirectionality of eye movement respectively. But these are not enough to predict the subsequent recall performance.

The resultant networks were further evaluated by the recall procedure, where a CA3 unit of the top layer (with overlap of 4) is initially activated and subsequent activity propagation in CA3 units according to recurrent connections is evaluated. Figure 2i shows the result of the recall activation that occurs in the time scale of theta cycle. The temporal evolution of the activity propagation is plotted in the equivalent graph shown in Figure 2h, where the color of the node indicates the peak activation time of each node unit. First the ‘star’ object unit and related scene units are activated (shown in green color), followed by the ‘sector’ object unit and related scene units (shown in red color) and finally the ‘cross’ object unit and related scene units (shown in dark red color). This recall sequence consisting of multiple object–place associations reflects the whole associative network structure through dynamics of the excitation of the recurrent connection and the global inhibition of the unit activation.

The mean number of correctly recalled object–place associations was 1.74 (s.d. 0.78), significantly smaller than the subject’s recall performance (mean recalled associations is calculated as 3.31 (s.d. 1.07)). However, the computational recall was significantly correlated with subjects’ subsequent recall performance (r = 0.1583, Z = 3.0811, p = 0.0021<0.01) that is larger than the correlation between the hierarchical connection weight sum, 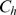, and subjects’ recall performance. This result successfully indicates that theta phase precession dynamics could predict the human subsequent recall performance.

### 2.2 Subsequent memory analysis by traditional predictors

In this section, we evaluate the correlation between experimental data during encoding and the subsequent memory performance. This analysis would reveal the cause of the significant correlation between computational and subjects’ memory recalls shown in the above section. First, as an index of subjects’ attentional state, the saccade number, 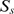, and the blink number, 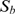, were evaluated. Both predictors were not found to significantly correlate with subjects’ recall performance (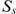: r = 0.0915, p = 0.0773; n.s., 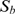: r = −0.0803. p = 0.1193; n.s.). Along with the result of connection weights sum, , that is dominantly related to the total fixation duration, simple behavioral parameters that did not include the spatial property of eye movement were unable to predict subjects’ recall.

Second, the mean object distance, *D*, was not significantly correlated to the subject’s recall performance (r = −0.0775, p = 0.1458; n.s.). *D* is an important parameter for the structure of the associative network, while this result shows that the object arrangement is not a dominant factor in the prediction of subsequent recall.

Finally, EEG theta power, 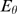 and EEG theta–saccade rate coherence, 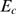, were evaluated. Both predictors significantly correlate with subjects’ memory recall (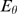: r = 0.1226, p = 0.0178<0.05; 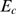: r = 0.1250, p = 0.0164<0.05) [23], [26]. The EEG theta modulation, that gates 50% of the input sequence, is not a cause of the significant correlation between computational and subjects’ recalls, because significant correlation was still obtained without EEG theta modulation (data not shown). It is rather considered that the eye movement pattern during high EEG theta was specifically associated with the memory formation. These results indicate that the model of theta phase precession produces a better predictor of subsequent recall than the traditional experimental predictors.

### 2.3 Clustering analysis among predictors

In order to clarify the relationship of 9 predictors (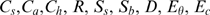), a hierarchical clustering using Ward’s method was applied. The resultant dendrogram is shown in Fig. 3b. Three clusters were found under the threshold of 0.7 maximum Euclidean distance. The first cluster (indicated by blue color) consisted of three model parameters (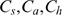) and saccade rate, 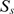. This cluster was characterized by eye movement patterns. The second cluster (indicated by green color) consisted of computational and subjects’ recalls (*R*, *C*) and the EEG theta–saccade rate coherence, 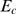. This result successfully demonstrates that the computational recall, *R*, is similar to subjects’ recall when compared with other indices. This second cluster is characterized by the interaction between eye movement and EEG theta. The last cluster (indicated by red color) consists of blink rate, 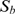, object spacing, *D*, and EEG theta power, 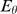, where 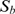 and 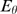 are considered to reflect the attentional state of the subject, and *D* could also be associated with the attentional effort. This cluster is considered to be related to the subjects’ attentional state. These results clearly indicate that the computational recall has similar information structure to subjects’ subsequent recall data as compared with other predictors.

### 2.4 Predictability of recall sequence

**Figure 4:**
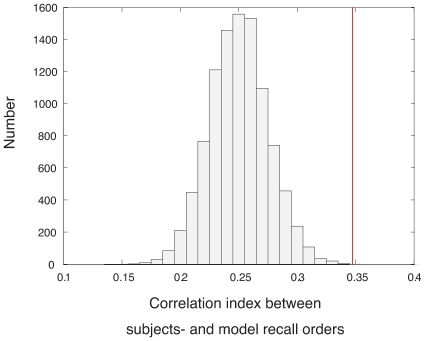
Correlation index between subjects’ and computational recall orders (red line) and distribution of correlation indices calculated under the condition of randomly shuffling recall order. The histogram bin is given by 0.01.

In addition to the prediction of subjects’ recall performance, we evaluated a relationship between subjects’ and computational recall orders that may further clarify the dynamic of theta phase precession. We calculated a correlation index between subjects’ and computational recall orders that we compared with correlation indices under a randomly shuffled recall order. Figure 4 shows the result of correlation indices where shuffling was repeated 10000 times. The correlation index between subjects’ and computational recall orders is significantly larger than the indices under the randomly shuffled condition (correlation index = 0.347, p = 0.0002). This result shows that the phase precession model predicts subjects’ recall order. Together with the significant correlation between subjects’ recall order and saccade sequence (p = 0.0010), the model may reflect the subjects’ encoding strategy represented in a saccade sequence.

### 2.5 Comparison with other models

**Figure 5:**
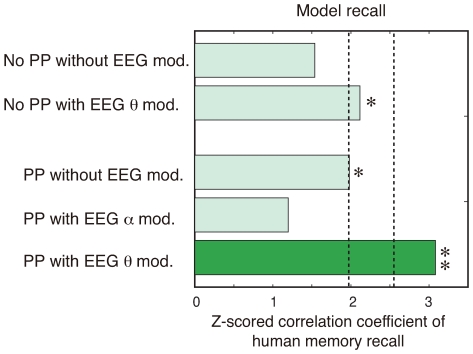
Z-score of correlation coefficient between subjects’ and computational recalls using different models where the effects of phase precession and EEG modulation are selectively removed. PP denotes ‘phase precession.’

The model used in the above analysis consists of multiple dynamics, such as phase precession and EEG theta modulation, therefore prompting the question of whether all dynamics are necessary for predicting subjects’ recall. To answer this question, we evaluated correlation coefficients between subjects’ and computational recalls by using different models where the effects of phase precession and/or EEG power modulation are selectively removed. In the results shown in Figure 5, the computational recall using the model with the same phase (no phase precession) and no EEG modulation is not significantly correlated with subjects’ recall (r = 0.0820, Z = 1.3579, p = 0.1745(n.s.)) On the other hand, the computational recall by the models with phase precession or EEG theta modulation were still significantly correlated with subjects’ recall (with phase precession; r = 0.1404, Z = 1.9798, p = 0.0477<0.05; with EEG theta modulation, r = 0.1109, Z = 2.1154, p = 0.0344<0.05). Moreover, the computational recall by the model with phase precession and EEG alpha modulation is not significantly correlated with subjects’ recall (r = 0.0787, Z = 1.1963, p = 0.2316(n.s.)). These results indicate that the combination of phase precession and EEG theta modulation is important for predicting subjects’ recall.

## Discussion

We demonstrated that the computational model of theta phase precession can predict subjects’ subsequent recall performance and that its predictive ability is better than experimental parameters (Fig. 3a) and other computational models (Fig. 5). Together with the clustering result of similarity between computational and subject’s recalls (Fig. 3b), the current finding showed that theta phase precession can exist in the human brain and produces a better predictor of the subsequent recall than other traditional predictors.

### 3.1 Components forming computational recall

Although the results successfully show that the computational recall could predict subjects’ subsequent recall (Fig. 3), it is still unclear why the computational recall could be a good index to predict subjects’ recall. In this section, we will discuss possible components differentiating the computational recall from other predictors. First, spatio-temporal patterns of eye movements dominantly determine the resultant associative network and the computational recall. The model consists of multiple spatial scales of scene units and stores the eye movement pattern in various spatial scales. These multi-scaled eye movement features characterize the subject’s strategic eye movement that would be related to the subject’s subsequent recall.

Second, the coherence between eye movement and EEG theta power also determines the resultant associative network. The eye movement does not always reflect the subject’s encoding state, but the EEG theta extracts the encoding-related eye movement. This is also supported by the results of the cluster analysis (Fig. 3b, second cluster), where the number of eye saccades appears in a different cluster.

Finally, the computational recall is a result of the simultaneous evaluation of the whole associative network formed during 8-sec encoding. This property is completely different from other computational predictors (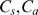 and 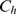). The associative network does not store individual object–place associations into separate networks but forms a cognitive map that represents a continuous space and object association. The computational recall appeared to reflect the hierarchical structure of the cognitive map with multiscaled scene information [28], thus the recall is an integrated value of memory encoding related activity within eye movement and EEG data.

### 3.2 Theta phase precession in humans

Theta phase precession pattern is in agreement with a time window of STDP, therefore it is expected to contribute to the synaptic plasticity, as suggested by Skaggs et al. [2]. Recently, theta phase precession is reported not only in the dentate gyrus, CA3 and CA1 regions [1], [2] but also in grid cells [29] in the entorhinal cortex [30]. The grid cells that are activated by the locations of regular triangular grid-like pattern and theta phase precession pattern also repeatedly appear. This pattern is also considered to contribute to the formation of place cells in the dentate gyrus [31].

Theta phase precession has not been reported in other species, while theta oscillation in the hippocampus has been considered to be important for the memory function commonly observed in many species. For example, bats show transient increase of theta LFP that would contribute to spatial cognition through echo-location [32], and the human hippocampus also shows transient LFP theta increase during navigation tasks [12], [14]. Theta oscillation in the hippocampus may be specifically related to the formation of environmental memory. Our current analysis suggests that theta phase precession exists in human brain.

### 3.3 Theory–experiment combined analysis

For the integrative understanding of the neural dynamics and the cognitive function, it is essential to combine both proposed computational models and evaluate them with experimental data. However, there are not many studies of human experimental data analysis with a specific computational model. Tanaka et al. proposed an interesting data analysis where human behavioral data of a Markov decision task were analyzed according to the temporal difference (TD) learning rule and applied for BOLD data analysis in human subjects [33]. This study clearly demonstrated that a specific neural dynamic, TD leaning rule with different time scales, exists in the human basal ganglia, and also demonstrated the ability of theory–experiment combined analysis to understand human neural dynamics.

In this paper, we used a theory–experiment combined analysis to elucidate theta phase precession in humans. The results successfully support the existence of theta phase precession and the resultant hierarchical cognitive map in humans. While it is still important to pay attention to ’epiphenomena’, i.e., it is still possible that ’true’ neural dynamics would have better computational ability and produce a consistent understanding of experimental data. To minimize this problem, the functional advantage of the computational model should validate the model in addition to the correlation between theory and experimental data. We have evaluated the functional advantage of theta phase precession [7], [8], [9], [15], but further studies are necessary.

## Methods

The computational model of theta phase precession [15], [28] is used to simulate the individual hippocampal network during object–place memory task in humans [23]. In this study, the computational predictors and traditional experimental predictors were compared with human subsequent recall.

### 4.1 Experimental data

The analyzed human experimental data were generated as part of a previous study [23]. Twelve volunteers participated in this experiment and performed an object-place association memory task with a recall test during EEG and eye movement recordings. Binocular eye movements were recorded with a video-based Eyelink I system (SR Research, Canada) with a 250 Hz sampling. Saccades and blinks were detected by a modified algorithm proposed by Engbert and Kliegl [34]. EEG signals were acquired using a Neuroscan amplifier (NeuroScan, Texas, USA). Ocular artifacts in the EEG were corrected by using the revised aligned-artifact average method developed by Croft et al. [35], where the horizontal electro-oculography (EOG), vertical EOG and radial EOG are linearly subtracted from raw EEG signal. Instantaneous frequency–energy characteristics for the corrected EEG were analyzed by convolution with complex Gaussian Morlet’s wavelet of which half-length is 6 [36].

Each subject participated in 36 trials. Each trial consisted of an 8-sec encoding phase of four objects randomly selected (subtended 0.42×0.42 degrees) and randomly located in a 3×3 grid square (5×5 degrees each), a 10-sec eye camera calibration task, and a recall test. During the encoding phase, the subjects memorized both object identities and these locations. During the recall test, the subjects were asked to reconstruct all object configurations on the display by using a mouse, where each object sequentially appears in a grid square by clicking of the mouse button. EEG and eye movement data of 350 artifact-free trials from eleven subjects during the encoding were used in the analysis.

### 4.2 Computational analysis of individual hippocampal network

The phase precession model of object–place memory [15], [28] was used to analyze the experimental data. The model consists of the visual input layer, the ECII (layer II in the entorhinal cortex) layer and the CA3 layer (Fig. 1a) where each layer consists of 9 object units and 39 scene units (Fig. 1b). In agreement with to the physiological evidence [37], [38], ECII units separately represent object and scene information that are combined in the CA3 layer. The object units characterize object features at the central visual field, and these are activated while fixating on a corresponding object at the center of the field. The scene units characterize a spatial distribution of eye fixations in the peripheral visual field. They have a receptive field in the peripheral vision and are activated when the eye fixation appears in the receptive field. These visual features are associated with the experimental stimulus. According to these visual input features, the human eye movement sequence is uniquely transformed to the input sequence of the model.

The ECII layer receives the input sequence and produces a theta phase precession pattern during high amplitude human EEG theta power in each trial. Phase precession in the entorhinal cortex has computational advantages in memory formation [7], [8], [9] that were recently supported by a rat experiment [30]. This simulates the intermittent increase of theta LFP in the hippocampus. EEG theta (at 7 Hz) at a central electrode (CP3) has a possible relationship with the hippocampal LFP theta [23], [24]. Theta phase precession is transmitted to the CA3 layer and is stored into connection weights according to the Hebb rule with an asymmetric time window. Detailed descriptions of the model are shown in Text S1. Additionally, EEG alpha at 11.5 Hz at a parietal electrode (P8) showing negative correlation to subjects’ subsequent recall [19] was used to evaluate the influence of EEG power modulation on the predictability of the model.

The model includes 16 parameters (two parameters in the input layer, 10 parameters in the ECII/CA3 layer and four parameters in the Hebb rule), but all parameters of the model were almost identical to the previous study [15], [28], except for input features. Time constants are key parameters for the memory formation and corresponded with rodent physiological evidence. The time window of the STDP is 12.5 ms [3], the theta period is 125 ms, and the duration of theta phase precession is 1 sec [1], [2]. This relationship, (asymmetric window of Hebb rule < theta cycle < duration of theta phase precession), is important to generate theta phase precession contributing to memory formation, and the small fluctuation of these parameters is not critical. Details of the parameters are described in Text S2.

#### 4.2.1 Computational memory encoding

Experimental data of each subject and trial were separately introduced to the computational model, i.e., experimental data including 350 trials of encoding and recall were separately analyzed. The initial condition of connection weight is given by and the model receives an 8-sec input sequence given by one trial experimental data of saccade and EEG during encoding. The resultant network after an 8-sec encoding period was used to predict subject’s subsequent recall performance. Three predictors of human subsequent recall were calculated, and each predictor included 350 values in relation to experimental trial number. The first predictor is the connection weight sum, 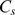, that is related to a total increase of connection weights. 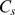 is given by
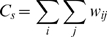
where 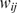 is the sum of the connection weight from the *j*-th to the *i*-th CA3 units. 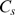 does not evaluate the unidirectional connections, so this is independent to the theta phase precession dynamics. The second predictor is an asymmetric connection weight sum, 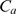, that is related to a total amount of asymmetric connection weights. 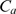 is given by
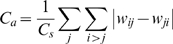


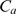
 and evaluates the amount of unidirectional connections formed by theta phase precession, while its information structure is not estimated. The third predictor is the hierarchical connection weight sum, 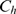, that is related to the formation of a cognitive map including object–place associations. 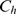 is given by
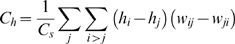
where 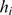 denotes the number of objects that could activate the *i*-th unit. For example, if the *i*-th unit is activated while fixating on object 1, 2 or 3 and is not activated while fixating on object 4, 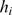 is 3. In the following section, 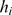 is termed an overlap of the *i*-th unit. 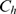 evaluates both the unidirectional connections and its information structure in terms of a hierarchical structure of the object-place memory. The above three predictors would characterize the associative network reflecting the spatio-temporal properties of subjects’ eye movement and EEG theta power.

#### 4.2.2 Computational memory recall

The resultant 350 units network was further evaluated by using a computational recall procedure. In the recall procedure, a scene unit with a receptive field covering all grids (with unit number 45) was activated initially. Then other CA3 units were automatically activated according to the stored recurrent connections. The global inhibition of the CA3 activation further supports the sequential activation of the CA3 units. The recalled sequence was decoded by using input visual features in terms of temporal coding. The number of successfully recalled object-place associations, *R*, was used as a predictor of subject’s subsequent recall. Equations and parameters of the recall procedure are described in Text S2.

### 4.3 The traditional experimental predictors

In addition to the four predictors calculated from the model, the traditional experimental predictors were also introduced. From eye movement data, saccade rate, 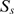, and blink rate, 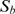, were calculated. The mean distance among objects, *D*, was also calculated as a stimulus parameter, by
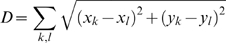
where *x* and *y* denote horizontal and vertical location of the fixated grid square. Furthermore, EEG theta power [23] and EEG theta power (at frontal midline (Fz) electrode)–saccade rate coherence [26] are already known to predict subsequent memory recall. EEG theta power, 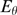, and a coherence between EEG theta and saccade rate, 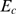, were also introduced.

### 4.4 Statistical procedure

The following three analyses were used to evaluate the ability of theta phase precession dynamics to predict human memory recall performance. In the first analysis (Section 3.1), the computational–predictors were calculated (as described in Section 4.2) and their prediction abilities were evaluated. For each predictor, the Spearman’s rank–sum correlation with subjects’ recall performance was calculated for individual subjects, and then the correlation coefficients of all subjects were integrated by using random effect analysis [39]. The difference from 0 (no correlation) was estimated by using the Z–test. In the second analysis (Section 3.2), the prediction abilities of the traditional experimental predictors (shown in Section 4.3) were further evaluated and compared with the prediction ability of the computational predictors. In the third analysis (Section 3.3), to reveal the information components organizing the computational predictors, the interaction among the computational and experimental predictors was evaluated by using a clustering analysis with a traditional Ward’s method [40]. In the fourth analysis (Section 3.4), to show the relationship between subjects’ and computational recall order, a correlation index was calculated as follows,
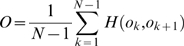
where 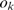 denotes the *k*-th object in the computational recall sequence and 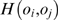 denotes a binary function showing the existence of ordered-pair 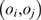 in subject’s recall (if the pair exists, then 1, otherwise 0). A mean correlation index of each trial is first calculated for individual subjects and then averaged over subjects weighted by the number of trials of each subject. The averaged correlation index is compared with correlation indices calculated under the condition of randomly shuffled recall order.

## Supporting Information

Text S1 The computational model of theta phase precession
(0.03 MB PDF)

Text S2 Model parameters and decoding
(0.02 MB PDF)
