# Supplementary material for: A Computational Predictor of Human Episodic Memory Based on a Theta Phase Precession Network
Source: PLoS One. 2009 Oct 23;4(10):e7536. doi: 10.1371/journal.pone.0007536 (PMC2762313; doi:10.1371/journal.pone.0007536)
Supplement: Text S1 — The computational model of theta phase precession (0.03 MB PDF) [file pone.0007536.s001.pdf]

Text S1: The computational model of theta phase precession

The computational model of the object-place memory based on theta phase coding [1] [2] consists of input layer, the ECII layer and the CA3 layer. All model parameters were identical to the previous study, except for the object and scene features in the input layer. The input layer consists of 9 object features and 36 scene features (Fig. 1b). According to the eye fixation location, the output of the i-th unit, I_i, is given by a binary value (0 or 1). For example, during a fixation at a left-upper grid square in Figure 1a, the PR unit representing object feature 4 and the PH units representing scene features 10, 19, 25, 31, 34 , 37, 41, 43, and 45 will be activated (indicated by 1) and the rest of the units stay at rest (indicated by 0). The dynamics of the ECII and CA3 layer units are described by the "phase model", where a single variable, the oscillation phase x(t) (mod 2π) can describe either neural oscillation or excitation [3, 4]. Output of the i-th unit with phase ϕ_i, p_i, is given by

$$p_i = \begin{cases} \cos(\phi_i) & (\cos(\phi_i) < 0), \\ 0 & (otherwise). \end{cases}$$

i-th ECII unit oscillation is activated by input, I_i, and phase coding is generated by phase locking with the theta rhythm of the local field potential, $\cos(\omega_0 t)$. It is assumed that the native frequency, ω_i, gradually increases, dependent on the input, I_i. The dynamics of ϕ_i^{ECII} are given by,

$$\dot{\phi_i^{\mathrm{ECII}}} = \omega_i - \left(C_{\mathrm{exc}}^{\mathrm{ECII}} I_i + C_{\mathrm{theta}}^{\mathrm{ECII}} \cos(\omega_0 t) - C_0^{\mathrm{ECII}}\right) \sin(\phi_i^{\mathrm{ECII}})$$

with

$$\tau_\omega \dot{\omega_i} = \omega_{\mathrm{L}} + (\omega_{\mathrm{H}} - \omega_{\mathrm{H}}) I_i - \omega_i$$

where w_0 is the angular frequency of the theta oscillation with period $T_0 = 2\pi/w_0$. This dynamic results in phase locking between the activation of the individual units and the theta rhythm. Phase precession is thereby realized in the output $p_i^{\mathrm{ECII}} = P(\phi_i^{\mathrm{ECII}})$.

The i-th CA3 unit accepts a single excitatory input from i-th ECII unit. The dynamics

of ϕ_i^{ECA3} is given by,

$$\dot{\phi_i^{\mathrm{CA3}}} = \omega_0 - \left(C_{\mathrm{exc}}^{\mathrm{CA3}} p_i^{\mathrm{ECII}} + C_{\mathrm{rec}}^{\mathrm{CA3}} \sum_{i \neq j} w_{ij} p_i^{\mathrm{CA3}} - C_{\mathrm{inh}}^{\mathrm{CA3}} \sum_i p_i^{\mathrm{CA3}} - C_0^{\mathrm{CA3}} \right) \sin(\phi_i^{\mathrm{CA3}})$$

where $C_{\mathrm{rec}}^{\mathrm{CA3}}$ and $C_{\mathrm{inh}}^{\mathrm{CA3}}$ denotes recurrent connections and the global inhibition, respectively.

In the memory encoding stage, the connection weights w_{ij} are determined using a modified Hebb rule with the STDP. For convenience the time evolution of w_{ij} is described by a delta function as,

$$\tau_w \dot{w_{ij}} = p_i^{\mathrm{CA3}}(t) p_j^{\mathrm{CA3}}(t - T_w) - C_{\mathrm{r}} p_i^{\mathrm{CA3}}(t - T_w) p_j^{\mathrm{CA3}}(t) - \left(p_i^{\mathrm{CA3}}(t) + p_j^{\mathrm{CA3}}(t) \right) w_{ij}$$

where τ_w and T_w are the time constants of w_{ij} and the time window of the STDP, respectively.

References

[1] Sato N, Yamaguchi Y (2005) On-line formation of a hierarchical cognitive map for object-place association by theta phase coding, Hippocampus 15: 963–978.

[2] N Sato, Y Yamaguchi (2009) Spatial-area selective retrieval of multiple object-place associations in a hierarchical cognitive map formed by theta phase coding. Cogn Neurodyn 3(2): 131–140.

[3] Hoppensteadt FC (1986) An introduction to the mathematics of neurons. Cambridge University Press, Cambridge, U.K. 192 p.

[4] Yamaguchi Y (1996) Synchronization of theta activities as a supervisory mechanism of the memory formation in a neural network model of the hippocampus. In: Kato N, editor. Functions and Clinical Relevance of the Hippocampus: Elsevier. pp. 339–344.
