# Supplementary material for: A Computational Predictor of Human Episodic Memory Based on a Theta Phase Precession Network
Source: PLoS One. 2009 Oct 23;4(10):e7536. doi: 10.1371/journal.pone.0007536 (PMC2762313; doi:10.1371/journal.pone.0007536)
Supplement: Text S2 — Model parameters and decoding (0.02 MB PDF) [file pone.0007536.s002.pdf]

Text S2: Model parameters and decoding

The computer experiment includes two stages, the memory encoding stage and the recall stage. The equations explained above are numerically calculated using the Runge-Kutta-Gill method. During encoding, the dynamics of the CA3 layer is assumed to be dominated by the dynamics of the ECII layer, simply as $p_i^{\mathrm{CA3}} = p_i^{\mathrm{ECII}}$. The initial state of connection weights is given by 0 ($w_{ij}(0) = 0$). The model parameters are identical to a previous study [1] ($C_{\mathrm{exc}}^{\mathrm{ECII}} = 3, C_{\mathrm{theta}}^{\mathrm{ECII}} = 1, C_0^{\mathrm{ECII}} = 3, tau_w = 0.1T_0, T_w = 100T_0, C_{\mathrm{r}} = 0.5$).

During recall, the dynamics of the ECII layer is assumed to be dominated by the input layer, simply as $p_i^{\mathrm{ECII3}} = I_i$, and connection weight, w_{ij} is assumed to be fixed ($w_{ij} = 0$). The model parameters were similar to previous study [1] ($C_{\mathrm{exc}}^{\mathrm{CA3}} = 1.5, C_{\mathrm{rec}}^{\mathrm{CA3}} = 3, C_{\mathrm{inh}}^{\mathrm{CA3}} = 0.1, C_0^{\mathrm{CA3}} = 1.5$). The recall is initiated by a rapid (100 ms) activation in the input unit that is classified as 4 object overlap (the top of the hierarchical network). The initial activation propagates in the CA3 network according to recurrent connections, and the sequence is decoded by corresponding object and scene features. In the decoding process, activation peak time, t_1, of each object unit is detected, then the corresponding position is decoded by the scene unit.

$$x, y : \frac{\sum_{j=10}^{45} G_j^{xy} p_j^{\mathrm{CA3}}(t_1)}{\sum_{j=10}^{45} G_j^{xy}} \rightarrow \max$$

where G_j^{xy} indicates the receptive field of the j-th scene input unit at location x, y (0: out of receptive field, 1: inside of receptive field).

References

[1] N Sato, Y Yamaguchi (2009) Spatial-area selective retrieval of multiple object-place associations in a hierarchical cognitive map formed by theta phase coding. Cogn Neurodyn 3(2): 131–140.
